# Metabolic Engineering and Adaptive Evolution for Efficient Production of l-Lactic Acid in Saccharomyces cerevisiae

**DOI:** 10.1128/spectrum.02277-22

**Published:** 2022-11-10

**Authors:** Pan Zhu, Rui Luo, Yize Li, Xiulai Chen

**Affiliations:** a State Key Laboratory of Food Science and Technology, Jiangnan University, Wuxi, China; b Key Laboratory of Industrial Biotechnology, Ministry of Education, Jiangnan University, Wuxi, China; c State Key Laboratory of Membrane Biology, School of Life Sciences, Tsinghua University, Beijing, China; Ocean University of China

**Keywords:** metabolic engineering, adaptive evolution, l-lactic acid, *Saccharomyces cerevisiae*

## Abstract

l-Lactic acid (LA) is a three-carbon hydroxycarboxylic acid with extensive applications in food, cosmetic, agricultural, pharmaceutical, and bioplastic industries. However, microbial LA production is limited by its intrinsic inefficiency of cellular metabolism. Here, pathway engineering was used to rewire the biosynthetic pathway for LA production in Saccharomyces cerevisiae by screening heterologous l-lactate dehydrogenase, reducing ethanol accumulation, and introducing a bacterial acetyl coenzyme A (acetyl-CoA) synthesis pathway. To improve its intrinsic efficiency of LA export, transporter engineering was conducted by screening the monocarboxylate transporters and then strengthening the capacity of LA export, leading to LA production up to 51.4 g/L. To further enhance its intrinsic efficiency of acid tolerance, adaptive evolution was adopted by cultivating yeast cells with a gradual increase in LA levels during 12 serial subcultures, resulting in a 17.5% increase in LA production to 60.4 g/L. Finally, the engineered strain S.c-NO.2-100 was able to produce 121.5 g/L LA, with a yield of up to 0.81 g/g in a 5-L batch bioreactor. The strategy described here provides a guide for developing efficient cell factories for the production of the other industrially useful organic acids.

**IMPORTANCE** Saccharomyces cerevisiae is one of the most widely engineered cell factories for the production of organic acids. However, microbial production of l-lactic acid is limited by its intrinsic inefficiency of cellular metabolism in S. cerevisiae. Here, the transmission efficiency of the biosynthetic pathway was improved by pathway optimization to increase l-lactic acid production. Then, the synthetic ability for l-lactic acid was further enhanced by adaptive evolution to improve acid tolerance of S. cerevisiae. Based on these strategies, the final engineered S. cerevisiae strain achieved high efficiency of l-lactic acid production. These findings provide new insight into improving the intrinsic efficiency of cellular metabolism and will help to construct superior industrial yeast strains for high-level production of other organic acids.

## INTRODUCTION

L-Lactic acid (LA) is a three-carbon hydroxycarboxylic acid with extensive applications in food, cosmetic, agricultural, pharmaceutical, and bioplastic industries (1, 2). Current industrial LA fermentations are based on different species of LA bacteria (3), but these bacteria are sensitive to low pH, and large amounts of neutralizing agents such as CaCO_3_ and NaOH are necessary for industrial LA production (4). Thus, LA production with LA bacteria is limited by its high production cost due to the regeneration of precipitate lactate salts (5). Thus, yeast is an attractive alternative for production of LA, due to its advantages such as growing and surviving in low pH. Various yeast species have been metabolically engineered for LA production, such as Saccharomyces cerevisiae (4, 6, 7), Kluyveromyces lactis (8, 9), Pichia stipitis (10), Zygosaccharomyces bailii (11), Candida utilis (12), and Candida boidinii (13). Among these, S. cerevisiae was the most widely engineered for LA production.

Six metabolic engineering strategies have been investigated for LA production in S. cerevisiae (Table 1). The first is to introduce heterologous lactate dehydrogenase (LDH) genes to redirect carbon flux from pyruvate to LA. When LDH from Lactobacillus plantarum and monocarboxylate transporters (JEN1) were overexpressed in S. cerevisiae, LA yield showed a large increase, to 0.52 g/g (14). The second strategy is to delete pyruvate decarboxylase genes (*PDC1*, -*5*, and -*6*) or alcohol dehydrogenase genes (*ADH1* to -*5*) to reduce ethanol accumulation. When the PDC1 and ADH1 genes were deleted, LA yield was significantly improved, to 0.75 g/g (15). The third strategy is to screen highly acid-tolerant yeasts to maintain a neutral intracellular pH. Based on the hypothesis that the better LA-producing strain has a higher intracellular pH, the high-LA-producing strain S. cerevisiae CEN.PK m850 was obtained by three consecutive rounds of cell sorting from the UV-mutagenized populations of S. cerevisiae Z26, and its LA production was increased to 70 g/L (16). In addition, by adaptive laboratory evolution of the LA-producing S. cerevisiae SR8LDH, the evolved S. cerevisiae BK01 was able to produce 119 g/L LA without the use of pH neutralizers (17). The fourth strategy is to express monocarboxylate transporters (JEN1, ADY2, or ESBP6) to export LA. The *JEN1* and *ADY2* genes were constitutively expressed in S. cerevisiae *jen1*Δ-LDH and S. cerevisiae *ady2*Δ-LDH, respectively, leading to a higher external LA concentration (5). The fifth strategy is to delete the *S*-adenosylmethionine synthetase (SAM2) gene to remodel the cell membrane during acid stress. When *SAM2* was deleted in S. cerevisiae CEN.PK m850, LA production was increased by 5.4%, to 69.2 g/L, compared with no *SAM2* deletion (18). The sixth strategy is to delete NADH-consuming enzymes (NDE1/2) to enhance the cofactor availability of intracellular redox. LA was produced at 117 g/L, with a yield of up to 0.58 g/g, under low-pH conditions by deleting *NDE1* and *NDE2* in S. cerevisiae SP3 (19). In summary, LA production has been improved by metabolic engineering strategies (20), but LA productivity still needs to be enhanced to improve the intrinsic efficiency of cellular metabolism.

**TABLE 1:** Comparison of LA production by S. cerevisiae strains

S. cerevisiae strain	Titer (g/L)	Yield (g/g glucose)	Productivity (g/L/h)	Reference
SPP	17.4	0.30	0.15	4
PK27	37.9	0.66	0.79	48
YIBO-7A	55.6	0.62	0.77	21
CEN.PK m850 *sam2Δ*	69.2	0.88	0.96	18
Z26	70	0.93	1.00	16
AF297C	75	0.75	0.75	15
YIBL-*pdc1/5Δ*	82.3	0.38	0.81	22
SP7	117	0.58	2.39	19
BK01	119	0.72	1.24	17
SP1130	142	0.89	3.55	24
NO.2-100	121.5	0.81	1.69	This study

**FIG 1:**
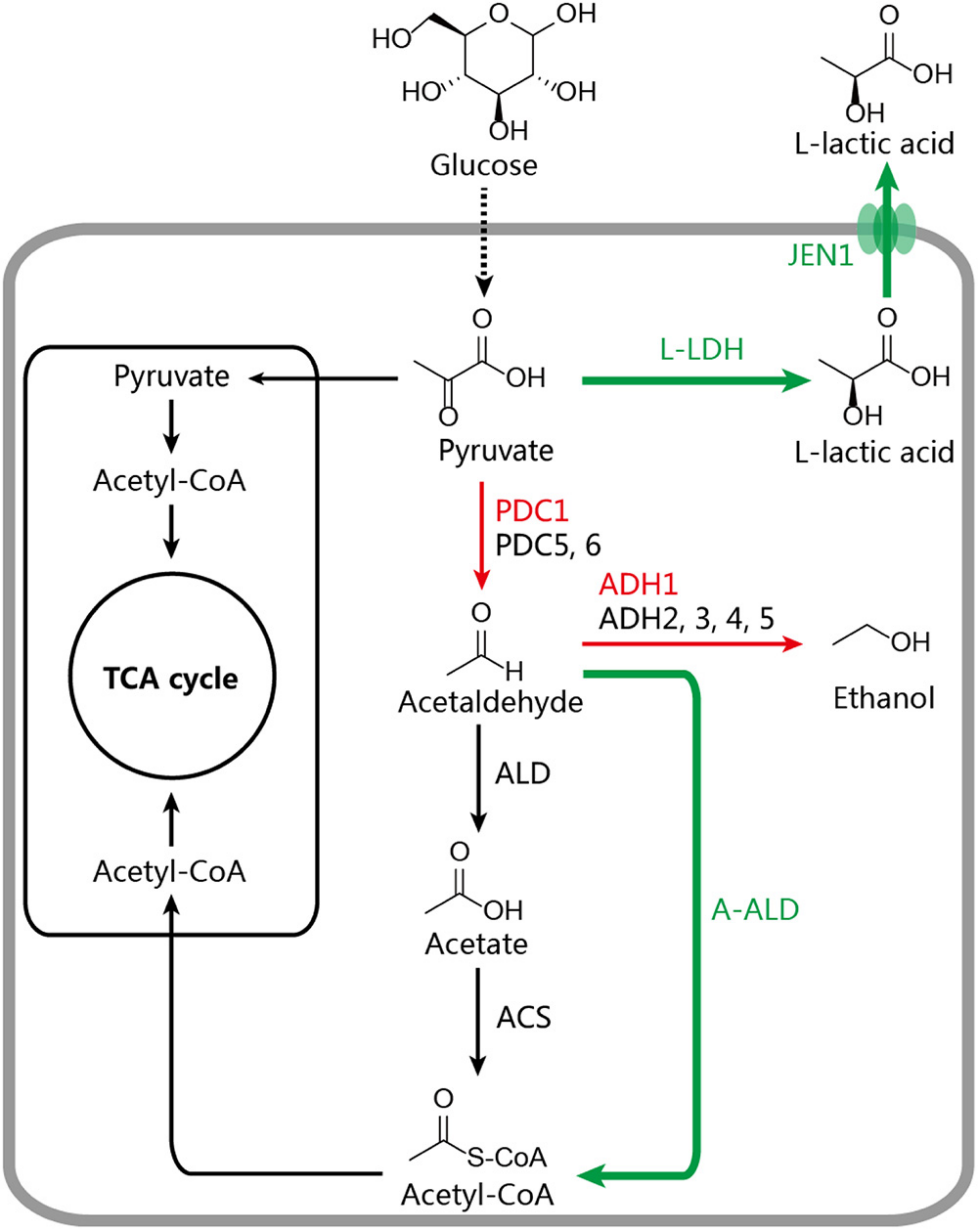
Major metabolic pathways for the formation of LA in S. cerevisiae. PDC1, 5, and 6, pyruvate decarboxylase; ADH1, 2, 3, 4, and 5, alcohol dehydrogenase; ALD, acetaldehyde dehydrogenase; ACS, acetyl-CoA synthetase; A-ALD, acetylating acetaldehyde dehydrogenase; l-LDH, l-lactate dehydrogenase; JEN1, monocarboxylate transporter; TCA, tricarboxylic acid.

In this study, S. cerevisiae was used as a model system to rewire the biosynthetic pathway for LA production (Fig. 1). Transporter engineering was conducted to improve LA export, and adaptive evolution was used to enhance acid tolerance. Based on these strategies, LA productivity was improved, and the final engineered strain, S.c-NO.2-100, was able to produce 121.5 g/L LA.

## RESULTS AND DISCUSSION

### Rewiring the biosynthetic pathway for LA production.

**FIG 2:**
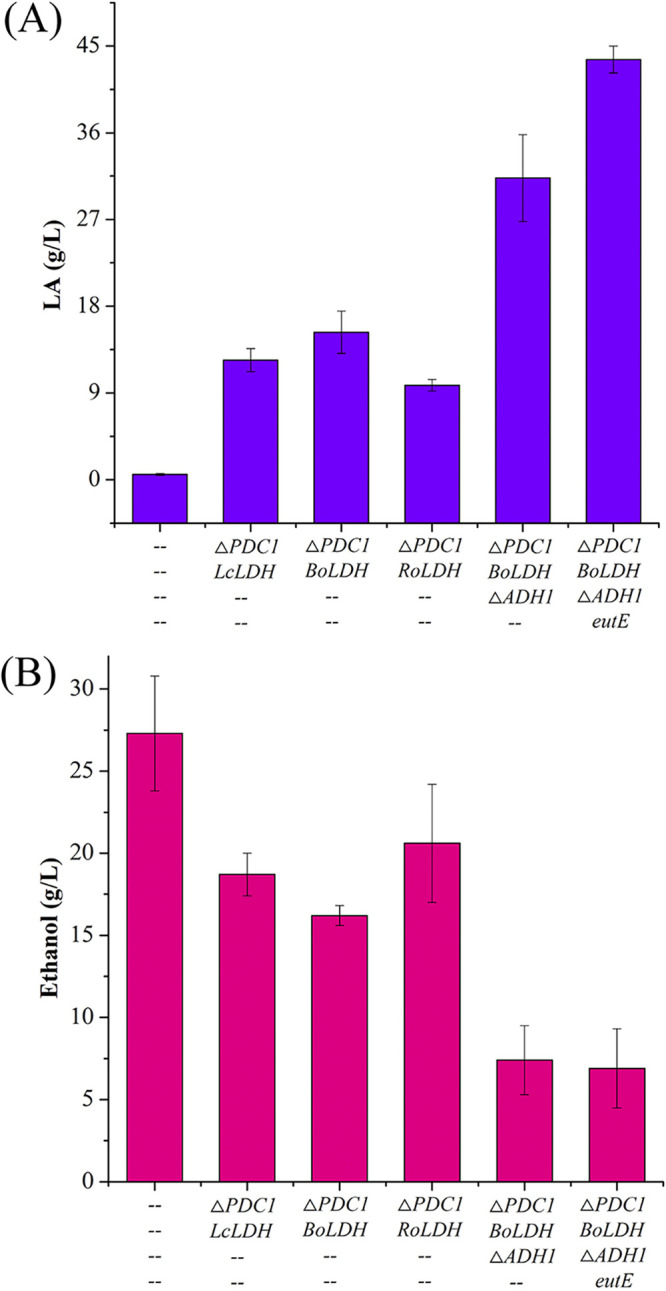
Rewiring the biosynthetic pathway for LA production. (A) Effect of gene expression or deletion on LA accumulation. (B) Effect of gene expression or deletion on ethanol accumulation. PDC1, pyruvate decarboxylase; ADH1, alcohol dehydrogenase; LDH, lactate dehydrogenase.

In S. cerevisiae, ethanol is the main by-product of l-lactic acid (LA) production (21). Three pyruvate decarboxylase (PDC) genes, *PDC1*, *PDC5*, and *PDC6*, contribute directly to ethanol production, but PDC activity is mainly from *PDC1* and *PDC5* genes (22). To enhance LA production and reduce ethanol accumulation simultaneously, three l-LDH genes, from Lactobacillus casei (*LcLDH*), bovines (*BoLDH*), and Rhizopus oryzae (*RoLDH*), were used to replace the coding region of *PDC1* in the chromosome of S. cerevisiae through homologous recombination. When *LcLDH*, *BoLDH*, and *RoLDH* were expressed, LA production was increased to 12.4 g/L, 15.3 g/L, and 9.8 g/L, respectively, which were 21.5-, 26.8-, and 16.8-fold higher than yields of the control strain S.c-0 (Fig. 2A). Ethanol accumulation was decreased by 31.5%, 40.7%, and 24.5%, but its titers were still as much as 18.7 g/L, 16.2 g/L, and 20.6 g/L, respectively (Fig. 2B). In addition, cell growth was reduced compared with that of the control strain S.c-0 (see Fig. S2 in the supplemental material), but there was no significant difference among *Lc*LDH, *Bo*LDH, and *Ro*LDH activities (Fig. S1). These results indicated that ethanol accumulation was not significantly reduced by deleting *PDC1*.

In S. cerevisiae, the cytosolic alcohol dehydrogenase (ADH1) gene contributes most of the catalytic activity for converting acetaldehyde to ethanol (15). To further reduce ethanol accumulation, we deleted the *ADH1* gene in strain S.c-PΔ-B. The resulting strain, S.c-PΔAΔ-B, produced only 7.4 g/L ethanol, which was 54.3% lower than that of strain S.c-PΔ-B (Fig. 2B). At the same time, the concentration of LA was increased by 104.6%, to 31.3 g/L, with its yield from glucose (Y_lac_) being 0.35 g/g (Fig. 2A). However, strain S.c-PΔAΔ-B showed growth retardation, leading to a 34.1% decrease in optical density at 600 nm (OD_600_) compared with that of strain S.c-PΔ-B (Fig. S2), possibly due to the accumulation of intracellular acetaldehyde caused by the *ADH1* deletion. This accumulation could affect the activity of acetaldehyde dehydrogenases (ALDs) by substrate inhibition (23), resulting in the deficient supply of acetyl coenzyme A (acetyl-CoA) for normal cell growth (24).

To overcome this limitation of the endogenous pathway, the heterogenous pathway was selected to replace or support the function of ALD–acetyl-CoA synthetase (ACS). The bacterially produced acetylating acetaldehyde dehydrogenase (A-ALD) can directly convert acetaldehyde to acetyl-CoA without energy consumption (25). Thus, the *eutE* gene from Escherichia coli was introduced and expressed under the control of the ADH1 promoter in strain S.c-PΔAΔ-B, resulting in strain S.c-PΔAΔ-BE. The specific activity of A-ALD in strain S.c-PΔAΔ-BE was increased by 2.1-fold compared with that of strain S.c-PΔAΔ-B (Fig. S3). In addition, cell growth of strain S.c-PΔAΔ-BE (OD_600_ = 10.4) was not significantly different from that of the control strain S.c-0 (Fig. S2). Further, LA titer (43.6 g/L) was increased by 39.3% compared with that of strain S.c-PΔAΔ-B (Fig. 2A). However, ethanol accumulation was similar with and without *eutE* expression (Fig. 2B). These results indicated that cell growth could be improved by introducing the heterogenous pathway to supply acetyl-CoA, increasing LA production.

The increased acetyl-CoA levels might serve as a driving force to increase the synthesis of acetyl-CoA-originated building blocks such as amino acids, fatty acids, and sterols (26). Based on this, cellular metabolic activities might be activated to redirect the major metabolic flux from ethanol accumulation to LA production (24).

### Improving LA production by transporter engineering.

**FIG 3:**
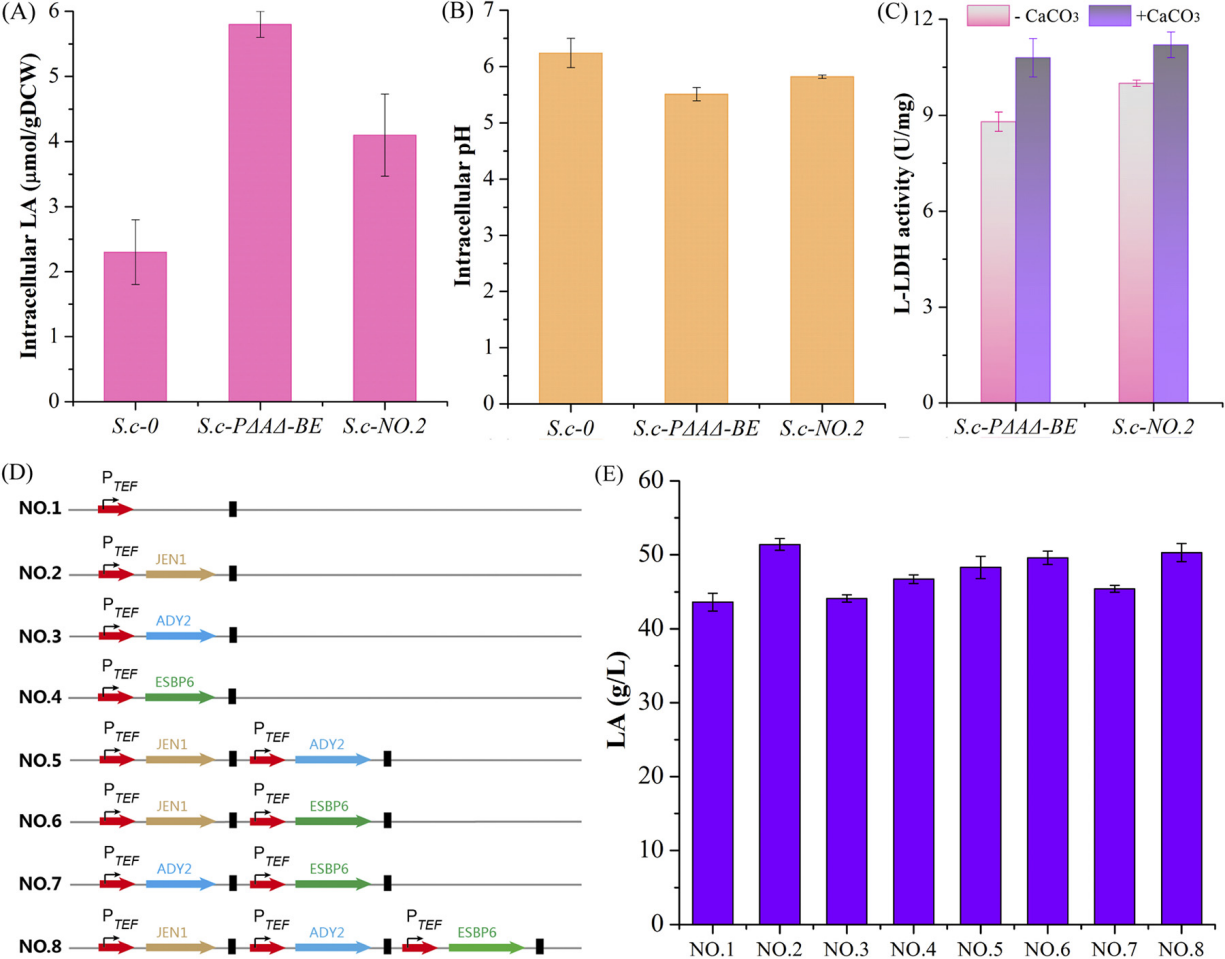
Improving LA production by transporter engineering. (A to C) Effects of the monocarboxylate transporters on intracellular LA concentrations (A), intracellular pH (C), and l-LDH activity. (D) A series of *JEN1*, *ADY2*, and *ESBP6* expression cassettes were designed with different combinations. (E) Concentrations of LA achieved by different *JEN1*, *ADY2*, and *ESBP6* expression cassettes.

Strain S.c-PΔAΔ-BE showed a large increase in LA production, but its Y_lac_ was only 0.48 g/g, possibly due to the fact that the accumulation of the intracellular LA causes intracellular acidification and l-LDH inhibition, leading to a decrease in Y_lac_ (14). To demonstrate this possibility, the intracellular LA and pH were determined for strains S.c-0 and S.c-PΔAΔ-BE. The concentration of intracellular LA in strain S.c-PΔAΔ-BE was increased by 152.2%, compared with that of the control strain S.c-0 (Fig. 3A). In addition, the intracellular pH in strain S.c-PΔAΔ-BE was 11.7% lower than that of the control strain S.c-0 (Fig. 3B). Furthermore, l-LDH activity in strain S.c-PΔAΔ-BE without CaCO_3_ as a neutralizing agent was reduced by 18.5% compared with that of CaCO_3_ addition (Fig. 3C). These results indicated that the accumulation of the intracellular LA exerted toxic effects on LA production, possibly suggesting that the transport capacity of LA needed to be enhanced to transport LA out of S. cerevisiae.

The genes *JEN1*, *ADY2*, and *ESBP6* encode the native monocarboxylate permeases, which have been used to export LA, acetic acid, formic acid (5, 27). First, we tested the effect of *JEN1*, *ADY2*, and *ESBP6* individually on LA production, and the highest concentration of LA (51.4 g/L) was obtained with strain S.c-NO.2 (Fig. 3D). At the same time, the intracellular LA was decreased by 29.3% compared with that of strain S.c-PΔAΔ-BE (Fig. 3A), and the intracellular pH was increased by 5.6% (Fig. 3B). In addition, its l-LDH activity without CaCO_3_ as a neutralizing agent was reduced by 10.3% compared with that seen with CaCO_3_ (Fig. 3C). Next, we analyzed the effect of combinations of two of these genes on LA production. When *JEN1* and *ESBP6* were overexpressed simultaneously, the LA titer increased to 49.6 g/L, which was similar to that of *JEN1* overexpression (Fig. 3D and E). Finally, the concentration of LA was increased to 50.3 g/L by simultaneously overexpressing *JEN1*, *ADY2*, and *ESBP6*, which was also similar to that of *JEN1* overexpression (Fig. 3D and E). These results indicated that the monocarboxylate transporters effectively enabled the export of LA, especially for JEN1.

JEN1 is a member of the sialate-proton symporter subfamily in the major facilitator superfamily (28). JEN1 can be induced to take up LA, but when LA is accumulated inside yeast cells (29), it also can mediate the efflux of LA (5, 14) due to the fact that the pK_a_ value of LA is much lower than the cytoplasmic pH value in yeast cells (14). Thus, a large proportion of the accumulated LA in cytosol is in the dissociated form and has to be actively transported outside yeast cells (14).

### Enhancing LA production by adaptive evolution.

**FIG 4:**
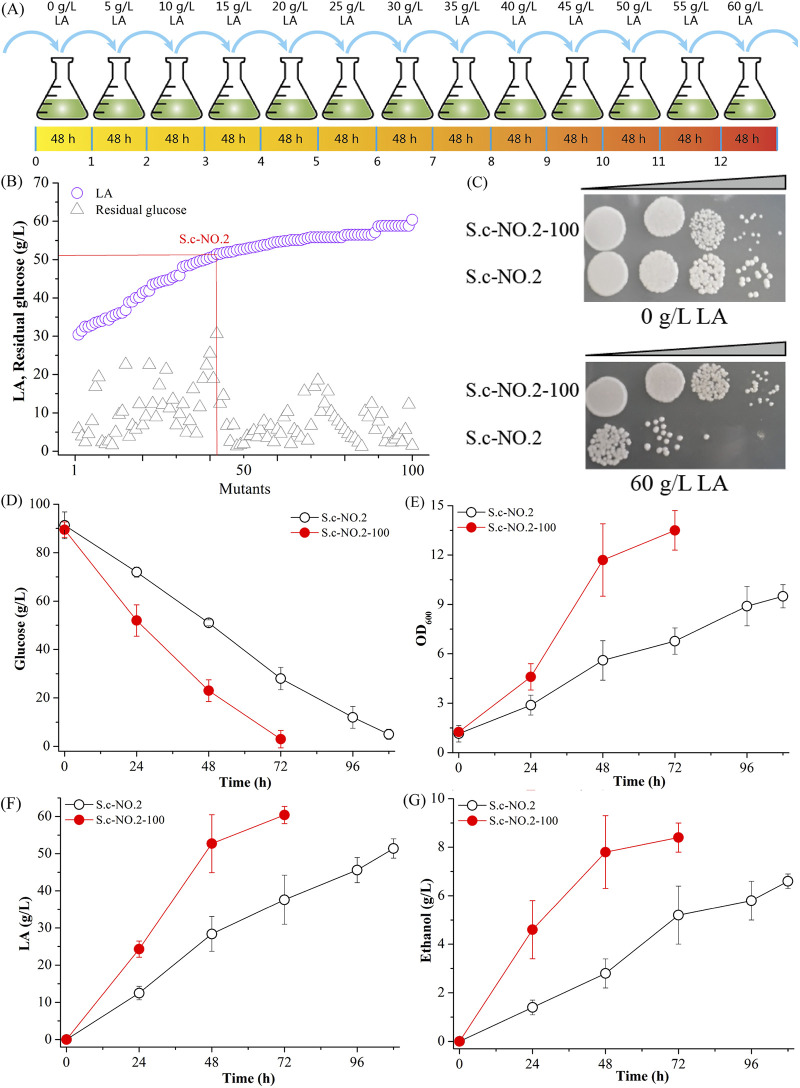
Enhancing LA production by adaptive evolution. (A) Schematic illustration of the adaptation process. (B to G) Effects of adaptive evolution on LA production (B), acid tolerance (C), glucose consumption (D), cell growth (E), LA production (F), and ethanol formation (G).

Although strain S.c-NO.2 showed good performance in LA production, growth limitation by the inhibitory effect of LA is still a major bottleneck for high production of LA (30). Thus, to further enhance LA productivity, we carried out adaptive evolution by cultivating the cells, with a gradual increase in LA levels from 10 to 60 g/L during 12 serial subcultures (Fig. 4A). Among 100 LA-tolerant candidate strains, strain S.c-NO.2-100 was selected based on glucose consumption ability and LA production level (Fig. 4B). In addition, strain S.c-NO.2-100 showed better growth than the unevolved strain S.c-NO.2 on medium A containing LA (Fig. 4C).

When the evolved strain S.c-NO.2-100 was used for LA fermentation, its final titer, yield, and LA production were increased to 60.4 g/L, 0.67 g/g, and 0.84 g/L/h, which are 17.5%, 17.5%, and 75.0% higher, respectively, than those of the unevolved strain S.c-NO.2 (Fig. 4D to G). In addition, its cell growth (OD_600_) and the average glucose consumption rate showed 42.1% and 50.6% increases compared with those of the unevolved strain (Fig. 4D and E). However, ethanol formation was increased by 27.2% compared with that of the unevolved strain (Fig. 4G). These results indicated that adaptive evolution was efficient for screening strains with good performance in the production of LA.

Adaptive evolution is a powerful tool for strain development in industrial applications (30, 31). Generally, adaptive evolution is performed by progressively increasing stress to screen microbes with the corresponding phenotype in batch cultivation, commonly by means of tube culture, flask culture, and plate culture (32). In this process, spontaneous mutations accumulate, thus yielding the desired phenotype (33). Further, multiple experimental purposes can be achieved by combining adaptive evolution with metabolic engineering, which have great potential in strain development. In this study, the evolved strain S.c-NO.2-100 was obtained, which showed good LA production and cell growth. However, the physiological mechanism underlying LA tolerance of S. cerevisiae still needs to be more thoroughly understood. Although LA tolerance appears to be a very complex trait, there is already substantial knowledge about this mechanism. The generation of LA tolerance has been demonstrated to be closely related to various cellular metabolism and regulation processes (34), as follows.

(i) The first such process is transcriptional regulation. The transcriptional response upon LA stress is largely regulated by the HAA1 regulon (35, 36). LA productivity was increased by overexpressing HAA1 in an LA-producing S. cerevisiae strain (31). (ii) Second is intracellular pH (pHi) homeostasis. pHi homeostasis is tightly regulated by the H^+^-ATPase pump (PMA1) in the plasma membrane and the V-ATPase pump in the vacuolar membrane (37). PMA1 overexpression was used as a candidate method for improving organic acids and low pH tolerance in yeast. (iii) The third process is anion transport. To counteract lactate anion accumulation in S. cerevisiae, anions have to be exported out of S. cerevisiae cells by lactate anion transporters such as JEN1 and ADY2. Overexpression of JEN1 and ADY2 could increase LA production in S. cerevisiae (5). (iv) The fourth process is reactive oxygen species (ROS) scavenging. ROS formed in S. cerevisiae under aerobic conditions not only can cause lipid, protein, and nucleic acid oxidative damage but also can act as second messengers to induce various cellular processes. To deal with this issue, S. cerevisiae could be metabolically engineered to increase the formation of ROS scavengers such as glutathione (GSH) (38) and ascorbic acid (39). (v) Next is cell envelope rearrangements. To counter weak acid stress or low external pH, cell envelope rearrangements can be achieved by reinforcing the cell wall structure to decrease porosity and altering the lipid composition of the plasma membrane to increase membrane rigidity (40, 41). As it is a key enzyme responsible for *S*-adenosylmethionine synthesis involved in phospholipid biosynthesis, deletion of SAM2 could further enhance acid tolerance and LA production in LA-producing S. cerevisiae (18). (vi) Last is amino acid, iron, and energy metabolism (34). LA stress can lead to a substantial decrease in intracellular amino acids by disrupting the proton gradient to affect the amino acid transporters and disturbing vacuolar integrity to affect amino acid storage in the vacuole. Metal cation homeostasis upon LA stress is regulated mainly by the transcription factor AFT1, which can alter the expression levels of many iron-related proteins. LA stress has a negative influence on energy metabolism through disruption of the electron transport chain, the ATP-generating metabolic pathways, and the energy-requiring export of protons and anions.

### Production of LA in a 5-L bioreactor.

**FIG 5:**
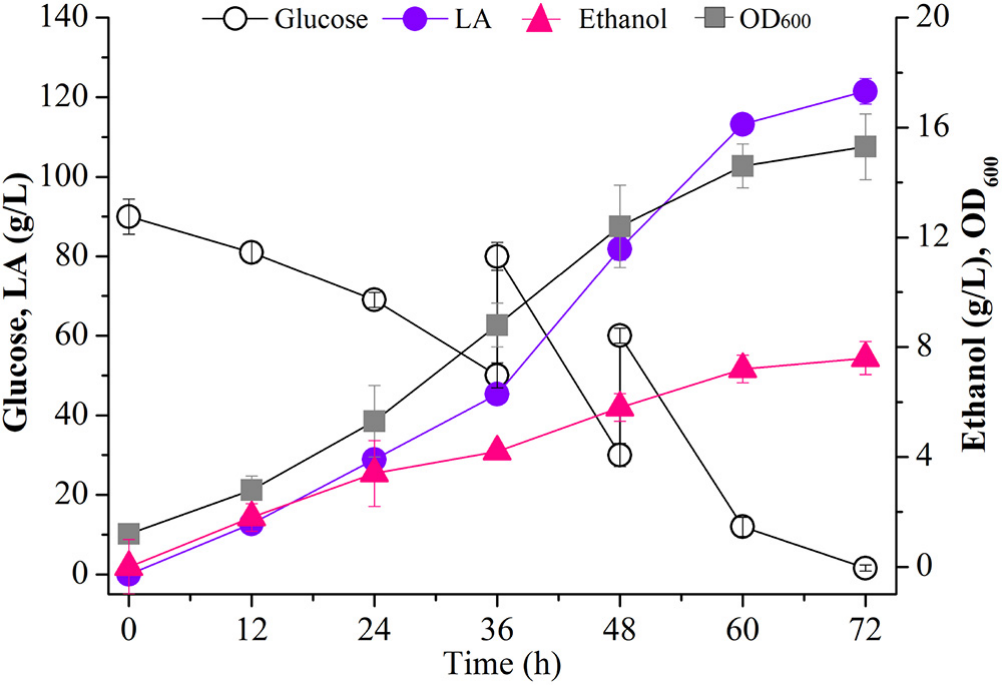
Production of LA by strain S.c-NO.2-100 in a 5-L batch bioreactor.

We next tested LA production of the evolved strain S.c-NO.2-100 in a 5-L batch bioreactor. In this batch culture, glucose was rapidly consumed during cell growth and LA synthesis and was depleted completely at 72 h (Fig. 5). Strain S.c-NO.2-100 grew continuously from 0 to 72 h and attained a maximal OD_600_ of 15.3 at 72 h (Fig. 5). LA accumulated gradually in the broth from 0 to 72 h, and the maximal titer, yield, and productivity of LA were 121.5 g/L, 0.81 g/g, and 1.69 g/L/h, respectively, at 72 h (Fig. 5). These results suggest that the final strain S.c-NO.2-100 can be utilized for efficient production of LA in fermentation.

### Conclusions.

In this study, the biosynthetic pathway for LA production was successfully rewired in S. cerevisiae by combining pathway construction with product transport. Then, the potential bottlenecks for LA production were rationally identified and removed by screening and strengthening the monocarboxylate transporters. Finally, the performance of the engineered strain in the production of LA was improved by adaptive evolution to promote cell tolerance to the high concentration of LA. Based on these strategies, the final concentration of LA with strain S.c-NO.2-100 was increased to 121.5 g/L. Although LA production with strain S.c-NO.2-100 in our study is lower than that of S. cerevisiae SP1130 in the previous study (24), our study offers an alternative strategy based on the combination of metabolic engineering and adaptive evolution. This strategy has great potential for developing efficient microbial cell factories for production of the other industrially useful organic acids.

## MATERIALS AND METHODS

### Strains and plasmids.

S. cerevisiae CEN.PK2-1C was used as the host strain for gene overexpression. The engineered S. cerevisiae strains used for l-lactic acid (LA) production in this study were derived from S. cerevisiae CEN.PK2-1C. E. coli JM109 and plasmid pY16 were used for plasmid construction. All strains and plasmids used in this study are listed in Table S1.

### DNA manipulation.

Gibson Assembly was used for plasmid construction according to the protocol of the Gibson Assembly cloning kit (New England Biolabs [NEB]). The l-lactate dehydrogenase (l-LDH) gene from bovines (*BoLDH*; GenBank ID D90141) was amplified from plasmid pLAZ10-LDH. The l-LDH gene from Lactobacillus casei FMME172 (*LcLDH*; gene ID 45549606) was amplified from the corresponding chromosomal DNA by PCR. The l-LDH gene from *Rhizopus oryzae* AS 3.381 (*RoLDH*; GenBank ID AAF74436.1) was amplified by PCR using the corresponding *c*DNA as the template. The aldehyde dehydrogenase gene (*eutE*; GenBank ID b2455) was PCR amplified from the Escherichia coli MG1655 genome. The monocarboxylate permease genes *JEN1* (GenBank ID YKL217W), *ADY2* (GenBank ID YCR010C), and *ESBP6* (GenBank ID YNL125C) were amplified from the *c*DNA of S. cerevisiae CEN.PK2-1C. The heterologous genes were inserted into the yeast genome together with appropriate auxotrophic marker genes.

### Medium.

Medium A, used for seed cultures, contained 1.95 g/L synthetic complete (SC) medium (Sunrise Science Products, catalog no. 1300-030) or SC-Ura (Sunrise Science Products, catalog no. 1306-030), 20 g/L glucose, 1.7 g/L yeast nitrogen base (YNB) (Sunrise Science Products, catalog no. 1500-100), and 5 g/L (NH_4_)_2_SO_4_.

Medium B, used for screening, contained 1.95 g/L SC-His (Sunrise Science Products, catalog no. 1303-030), SC-Leu (Sunrise Science Products, catalog no. 1304-030), or SC-Ura, 20 g/L glucose, 1.7 g/L YNB, and 5 g/L (NH_4_)_2_SO_4_.

Medium C, used for fermentation, contained 1.95 g/L SC or SC-Ura, 90 g/L glucose, 3.4 g/L YNB, and 5 g/L (NH_4_)_2_SO_4_.

### Culture conditions.

The seed culture was cultivated for 24 h on a reciprocal shaker (200 rpm) at 30°C in a 250-mL flask containing 25 mL medium A. Then, the broth was centrifuged, the supernatant liquid was discarded, and the pellet was suspended in fresh medium C. Next, the cell suspension was divided equally among 500-mL flasks containing 50 mL fresh medium C with an initial biomass OD_600_ of 1.5. This cell culture was buffered with 50 g/L CaCO_3_ and fermented at 30°C for 108 h with rotation at 200 rpm.

Batch fermentation was performed in a 5-L bioreactor New Brunswick Scientific Co., Inc., NJ, USA (NBS) containing 2.5 L medium C with an initial biomass OD_600_ of 1.5. Fermentation was performed at 30°C for 72 h with agitation at 200 rpm and aeration at 1.0 vvm. Culture pH was controlled at 5.5 using 8 mol/L NaOH. In batch fermentation, 30 g/L glucose was fed at 36 h and 48 h, but this process was not needed in shake flasks.

### Adaptive evolution.

To develop LA-tolerant strains, adaptive laboratory evolution was carried out by growing cells in medium A with a gradual increase in lactate concentration from 10 to 60 g/L during 12 subcultures. During the evolution, growth rate and metabolite titer were analyzed to identify the characterization of the evolved strains. After the final subculture, LA-tolerant colonies were isolated on solid medium A containing 60 g/L LA. Then, LA production and glucose consumption were tested. Among the evolved strains, the most efficient strain was selected.

### Intracellular pH measurement.

pHi was measured by analyzing fluorescence intensity with a spectrofluorophotometer with excitation at 430 and 490 nm and emission at 525 nm, after yeast cells were stained with the pH-sensitive probe 5(6)-carboxyfluorescein diacetate succinimidyl ester (CFDA-SE; Sigma-Aldrich, St. Louis, MO, USA) (42). Briefly, S. cerevisiae S.c-0, S.c-PΔAΔ-BE, and S.c-NO.2 were incubated in medium C for 48 h, and then S. cerevisiae cells were collected, washed, and resuspended in 50 mM citric/phosphate buffer (OD_600_ = 0.5). Next, CFDA-SE was added at a final concentration of 150 μM, and then the cell suspension was incubated at 30°C for 1 h. After removing the unloaded probe with citric/phosphate buffer, the fluorescence intensity was measured by spectrofluorophotometer. The intracellular pH could be calculated with the fluorescence intensity by a calibration curve. The calibration curve was plotted by incubating log-phase wild-type S. cerevisiae in 50 mM citric/phosphate buffer at pH 4.0 to 7.5 (0.5 unit per interval) with CFDA-SE. Carbonyl cyanide *m*-chlorophenyl hydrazone (0.5 mM, Sigma-Aldrich, St. Louis, MO, USA) was used to make the intracellular pH similar to the extracellular pH.

### Tolerance assay.

The growth of S. cerevisiae strains in log phase was diluted to an absorbance at 600 nm (OD_600_) of 1.0 in phosphate-buffered saline. Aliquots (4 μL) of 10-fold serial dilutions were spotted onto medium A plates with different concentrations of LA.

### Analytical methods.

The OD_600_ was assayed with a spectrophotometer. The concentrations of glucose, ethanol, and LA were determined by high-performance liquid chromatography (HPLC) as described in reference 43. Intracellular metabolites were extracted by freeze-thawing in methanol as described in reference 44. The intracellular level of LA was determined by HPLC according to the procedure described in previous reports (43).

### Enzyme activity assays.

Cell extracts were prepared for the determination of enzyme activity (45). The activity of l-LDH was determined by measuring the oxidation of NADH spectrophotometrically at 340 nm with the conversion of pyruvate to lactate (45). One unit of l-LDH activity was defined as the amount of enzyme required to convert 1 μmol of NADH to NAD^+^ per minute. A-ALD was assessed spectrophotometrically by monitoring the reduction of NAD^+^ to NADH at 340 nm (46). Protein concentrations in cell extracts were determined by the Lowry method (47).

### Data availability.

The data underlying this article are available in the article.
