# Supplementary material for: Metabolic Engineering and Adaptive Evolution for Efficient Production of l-Lactic Acid in Saccharomyces cerevisiae
Source: Microbiol Spectr. 2022 Nov 10;10(6):e02277-22. doi: 10.1128/spectrum.02277-22 (PMC9769770; doi:10.1128/spectrum.02277-22)
Supplement: Supplemental file 1 — Supplemental material. Download spectrum.02277-22-s0001.pdf, PDF file, 0.3 MB [file spectrum.02277-22-s0001.pdf]

Supplemental material

Metabolic Engineering and Adaptive Evolution for Efficient Production of L-lactic acid in *Saccharomyces cerevisiae*

Pan Zhu[a,c], Rui Luo[a,b], Yize Li[a,b], and Xiulai Chen[a,b]*

[a] State Key Laboratory of Food Science and Technology, Jiangnan University, Wuxi 214122, China;

[b] Key Laboratory of Industrial Biotechnology, Ministry of Education, Jiangnan University, Wuxi 214122, China;

[c] State Key Laboratory of Membrane Biology, School of Life Sciences, Tsinghua University, Beijing 100084, China

* Corresponding author at:

State Key Laboratory of Food Science and Technology, Jiangnan University, 1800 Lihu Road, Wuxi 214122, China. Tel: +86-510-85197357; Fax: +86-510-85197875. *E-mail address*: xlchen@jiangnan.edu.cn (Xiulai Chen)

Table S1 Strains and plasmids used in this study.

Strains and plasmids	Relevant characteristics	References
**Strains**		
S. cerevisiae CEN.PK2-1C	*MATa ura3-52 leu3-5,112 trp1-289 his3Δ MAL2-8^{c} SUC2*	EUROSCARF
S.c-0	*S. cerevisiae* CEN.PK2-1C	EUROSCARF
S.c-PΔ-L	*S. cerevisiae* CEN.PK2-1C *pdc1*Δ::P$_{PDC1}$-*LcLDH*-T::*his*	This study
S.c-PΔ-B	*S. cerevisiae* CEN.PK2-1C *pdc1*Δ::P$_{PDC1}$-*BoLDH*-T::*his*	This study
S.c-PΔ-R	*S. cerevisiae* CEN.PK2-1C *pdc1*Δ::P$_{PDC1}$-*RoLDH*-T::*his*	This study
S.c-PΔAΔ-B	S.c-PΔ-B *adh1*Δ::*leu*	This study
S.c-PΔAΔ-BE	S.c-PΔ-B *adh1*Δ::P$_{ADH1}$-*eutE*-T::*leu*	This study
S.c-NO.1	S.c-PΔAΔ-BE (pY16)	This study
S.c-NO.2	S.c-PΔAΔ-BE (pY16-JEN1)	This study
S.c-NO.3	S.c-PΔAΔ-BE (pY16-ADY2)	This study
S.c-NO.4	S.c-PΔAΔ-BE (pY16-ESBP6)	This study
S.c-NO.5	S.c-PΔAΔ-BE (pY16-JEN1-ADY2)	This study
S.c-NO.6	S.c-PΔAΔ-BE (pY16-JEN1-ESBP6)	This study
S.c-NO.7	S.c-PΔAΔ-BE (pY16-ADY2-ESBP6)	This study
S.c-NO.8	S.c-PΔAΔ-BE (pY16-JEN1-ADY2-ESBP6)	This study
S.c-NO.2-100	the evolved S.c-NO.2	This study
**Plasmids**		
pY16	CEN6/ARSH4, Amp, URA3, P$_{TEF}$	Turbo
pY16-JEN1	CEN6/ARSH4, Amp, URA3, P$_{TEF}$-JEN1	This study
pY16-ADY2	CEN6/ARSH4, Amp, URA3, P$_{TEF}$-ADY2	This study
pY16-ESBP6	CEN6/ARSH4, Amp, URA3, P$_{TEF}$-ESBP6	This study
pY16-JEN1-ADY2	CEN6/ARSH4, Amp, URA3, P$_{TEF}$-JEN1, P$_{TEF}$-ADY2	This study
pY16-JEN1-ESBP6	CEN6/ARSH4, Amp, URA3, P$_{TEF}$-JEN1, P$_{TEF}$-ESBP6	This study
pY16-ADY2-ESBP6	CEN6/ARSH4, Amp, URA3, P$_{TEF}$-ADY2, P$_{TEF}$-ESBP6	This study
pY16-JEN1-ADY2-ESBP6	CEN6/ARSH4, Amp, URA3, P$_{TEF}$-JEN1, P$_{TEF}$-ADY2, P$_{TEF}$-ESBP6	This study
pLAZ10-LDH	pLAZ10 with *LDH* gene from *Bovine*	(1)

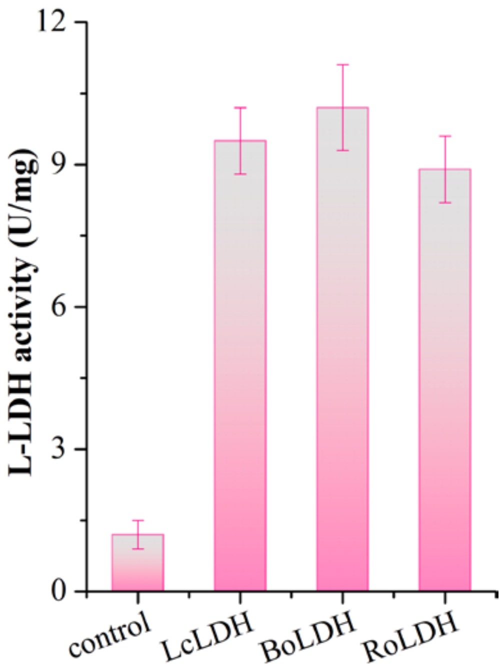

FIG S1 The specific activities of *Lc*LDH, *Bo*LDH, and *Ro*LDH in the engineered *S. cerevisiae*, S.c-PΔ-L, S.c-PΔ-B, and S.c-PΔ-R, respectively.

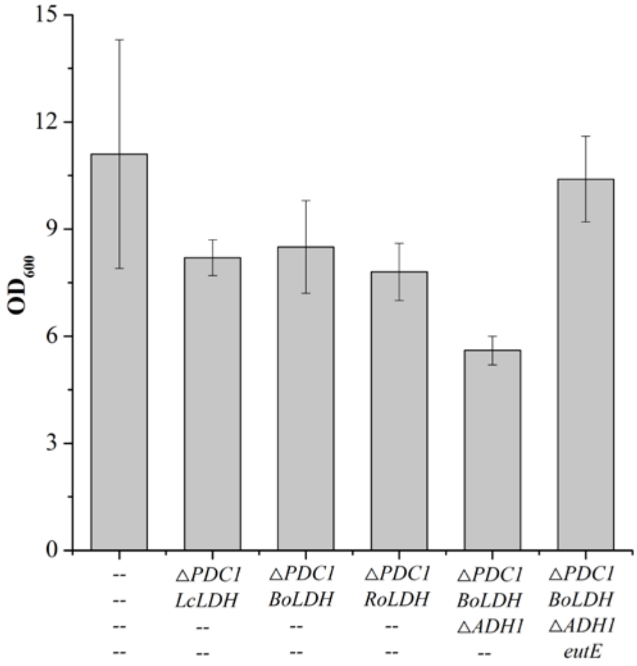

FIG S2 Effect of gene expression or deletion on cell growth.

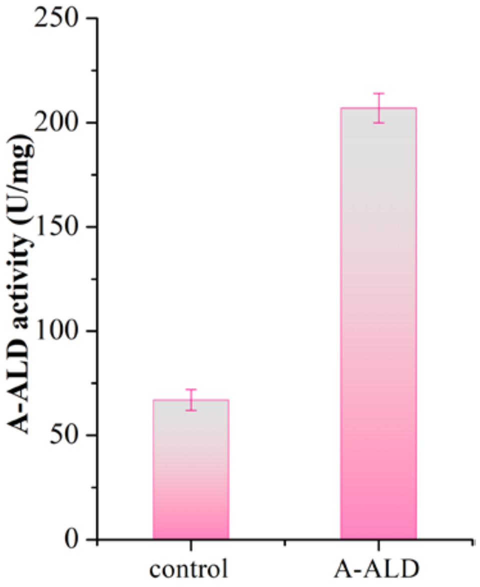

FIG S3 The specific activities of A-ALD in the engineered strain S.c-PΔAΔ-BE and the control strain S.c-PΔAΔ-B.

References

1. **Bianchi MM, Brambilla L, Protani F, Liu CL, Lievense J, Porro D.** 2001. Efficient homolactic fermentation by *Kluyveromyces lactis* strains defective in pyruvate utilization and transformed with the heterologous LDH gene. Appl Environ Microbiol **67:**5621-5625.
